# Supplementary material for: Ketoanalogue Supplementation in Patients with Non-Dialysis Diabetic Kidney Disease: A Systematic Review and Meta-Analysis
Source: Nutrients. 2022 Jan 19;14(3):441. doi: 10.3390/nu14030441 (PMC8838123; doi:10.3390/nu14030441)
Supplement: Supplementary file 1 [file nutrients-14-00441-s001.zip › nutrients-1537363-supplementary.pdf]

Supplementary materials to the manuscript: "**Ketoanalogue Supplementation in Patients with Non-dialysis Diabetic Kidney Disease: A Systematic Review and Meta-analyses"** by V. Bellizzi et al.

Online Supplement

Literature Search Protocols: Ketoanalogues in Diabetic Renal Failure

Aim: Identify published clinical studies reporting outcomes on patients with diabetic chronic kidney disease receiving supplemental ketoanalogues.

Databases searched: MedLine®, Embase™, the Cochrane Database of Systematic Reviews, the Cochrane Central Register of Controlled Trials, PubMed®, and Web of Science™

Search periods:

Cochrane Central Register of Controlled Trials: Issue 7 of 12, July 2020
Cochrane Database of Systematic Reviews: Issue 7 of 12, July 2020
Embase: 1947 – March 24, 2021
Medline: 1946 – March 24, 2021
PubMed: Jan 2020 – March 24, 2021
Web of Science: 1960 – March 24, 2021

Cochrane search strategy

#1 (keto-analogs or keto-analog):ti,ab,kw
#2 (keto-analogues or keto-analogue):ti,ab,kw
#3 (ketoanalogs or ketoanalog):ti,ab,kw
#4 (ketoanalogues or ketoanalogue):ti,ab,kw
#5 KA NEAR/2 EAA
#6 'ketoacid supplements':ti,ab,kw
#7 MeSH descriptor: [Keto Acids] explode all trees
#8 ketoacids:ti,ab,kw
#9 'keto acids':ti,ab,kw
#10 #1 or #2 or #3 or #4 or #5 or #6 or #7 or #8 or #9
#11 MeSH descriptor: [Renal Insufficiency, Chronic] explode all trees
#12 chronic kidney disease:ti,ab,kw
#13 CKD:ti,ab,kw
#14 advanced kidney disease:ti,ab,kw
#15 chronic renal insufficiency:ti,ab,kw
#16 renal failure:ti,ab,kw
#17 predialysis kidney disease:ti,ab,kw
#18 pre-dialysis kidney disease:ti,ab,kw
#19 #11 or #12 or #13 or #14 or #15 or #16 or #17 or #18
#20 MeSH descriptor: [Diabetes Mellitus] explode all trees
#21 diabetes:ti,ab,kw
#22 diabetic*:ti,ab,kw
#23 #20 or #21 or #22

#24 #19 and #23
#25 diabetic nephropath*:ti,ab,kw
#26 diabetic kidney disease:ti,ab,kw
#27 #24 or #25 or #26
#28 #10 and #27

Embase & Medline search strategy

1 (keto-analogs or keto-analog).ab,de,kw,ot,sh,ti,tw.
2 (keto-analogues or keto-analogue).ab,de,kw,ot,sh,ti,tw.
3 (ketoanalogs or ketoanalog).ab,de,kw,ot,sh,ti,tw.
4 (ketoanalogues or ketoanalogue).ab,de,kw,ot,sh,ti,tw.
5 (KA adj2 EAA).ab,de,kw,ot,sh,ti,tw.
6 (ketoacid* adj2 supplement*).ab,de,kw,ot,sh,ti,tw.
7 exp Keto Acids/
8 ketoacids.ab,de,kw,ot,sh,ti,tw.
9 'keto acids'.ab,de,kw,ot,sh,ti,tw.
10 1 or 2 or 3 or 4 or 5 or 6 or 7 or 8 or 9
11 exp Renal Insufficiency or exp Renal Insufficiency/
12 chronic kidney disease.ab,de,kw,ot,sh,ti,tw.
13 CKD.ab,de,kw,ot,sh,ti,tw.
14 chronic renal insufficiency.ab,de,kw,ot,sh,ti,tw.
15 (chronic or acute) adj2 'renal failure'.ab,de,kw,ot,sh,ti,tw.
16 chronic kidney failure.ab,de,kw,ot,sh,ti,tw.
17 chronic renal disease.ab,de,kw,ot,sh,ti,tw.
18 advanced kidney disease.ab,de,kw,ot,sh,ti,tw.
19 advanced renal disease.ab,de,kw,ot,sh,ti,tw.
20 predialysis kidney.ab,de,kw,ot,sh,ti,tw.
21 pre-dialysis kidney.ab,de,kw,ot,sh,ti,tw.
22 11 or 12 or 13 or 14 or 15 or 16 or 17 or 18 or 19 or 20 or 21
23 exp Diabetes Mellitus/
24 exp Diabetes/
25 diabetes.ab,de,kw,ot,sh,ti,tw.
26 diabetic*.ab,de,kw,ot,sh,ti,tw.
27 23 or 24 or 25 or 26
28 22 and 27
29 diabetic nephropath*.ab,de,kw,ot,sh,ti,tw.
30 diabetic kidney disease.ab,de,kw,ot,sh,ti,tw.
31 28 or 29 or 30
32 10 and 31
33 limit 32 to humans
34 (comment or editorial or letter or note or review).pt,sh.
35 33 not 34
36 remove duplicates from 35
37 exp Systematic Review/

38 (systematic* adj2 review*).ab,de,kw,ot,sh,pt,ti,tw.
39 exp Randomized Controlled Trials as Topic/
40 exp Meta-Analysis/
41 Meta-Analysis.pt.
42 exp Consensus/
43 Consensus.pt,sh.
44 exp practice guideline/
45 Guideline.pt,sh.
46 ('position paper' or 'position statement').ab,de,kw,ot,sh,pt,ti,tw.
47 37 or 38 or 39 or 40 or 41 or 42 or 43 or 44 or 45 or 46
48 36 and 47
49 from 48 keep 1-14
50 exp randomized controlled trials/ or exp randomized controlled trial/
51 randomized controlled trial.pt
52 exp random allocation/ or exp randomization/
53 exp Double-Blind Method/ or exp Double-Blind Procedure/
54 exp Single-Blind Method/ or exp Single-Blind Procedure/
55 exp placebos/ or exp placebo/
56 ((single or double or treble or triple) adj2 (blind* or mask* or umm*)).ab,de,kw,ot,sh,pt,ti,tw.
57 random*.ab,de,kw,ot,sh,pt,ti,tw.
58 blind*.ab,de,kw,ot,sh,pt,ti,tw.
59 parallel*.ab,de,kw,ot,sh,pt,ti,tw.
60 masked.ab,kw,ot,sh,ti,tw.
61 placebo*.ab,de,kw,ot,sh,pt,ti,tw.
62 assign*.ab,de,kw,ot,sh,pt,ti,tw.
63 50 or 51 or 52 or 53 or 54 or 55 or 56 or 57 or 58 or 59 or 60 or 61 or 62
64 36 and 63
65 64 not 49
66 from 65 keep 1-57
67 exp Clinical Trial/
68 clinical trial.ab,de,kw,ot,sh,pt,ti,tw.
69 clinical study.ab,de,kw,ot,sh,pt,ti,tw.
70 (Phase II adj3 trial).ab,de,kw,ot,sh,pt,ti,tw.
71 (Phase III adj3 trial).ab,kw,ot,sh,pt,ti,tw.
72 (Phase IV adj3 trial).ab,kw,ot,sh,pt,ti,tw.
73 (Phase II adj3 study).ab,de,kw,ot,sh,pt,ti,tw.
74 (Phase III adj3 study).ab,kw,ot,sh,pt,ti,tw.
75 (Phase IV adj3 study).ab,kw,ot,sh,pt,ti,tw.
76 exp major clinical study/
77 prospective study.ab,kw,ot,sh,pt,ti,tw.
78 exp Treatment Outcome/
79 exp Multicenter Study/
80 multicent*.ab,kw,ot,sh,pt,ti,tw.
81 exp Prospective Studies/

82 exp Retrospective Studies/
83 retrospective study.ab,kw,ot,sh,pt,ti,tw.
84 retrospective analysis.ab,kw,ot,sh,pt,ti,tw.
85 exp Follow-Up Studies/
86 Comparative Study.pt.
87 exp comparative study/
88 exp drug comparison/
89 controlled study.ab,de,kw,ot,sh,pt,ti,tw.
90 controlled trial.ab,de,kw,ot,sh,pt,ti,tw.
91 exp Observational Study/
92 open-label.ab,kw,ot,sh,pt,ti,tw.
93 pilot study.ab,kw,ot,sh,pt,ti.
94 exp Longitudinal Studies/
95 longitudinal study.ab,de,kw,ot,sh,ti,tw.
96 exp Case Series/
97 Case Reports.pt.
98 exp Case Reports/
99 67 or 68 or 69 or 70 or 71 or 72 or 73 or 74 or 75 or 76 or 77 or 78 or 79 or 80 or 81 or 82 or 83 or 84 or 85 or 86 or 87 or 88 or 89 or 90 or 91 or 92 or 93 or 94 or 95 or 96 or 97 or 98
100 36 and 99
101 100 not (49 or 66)
102 from 101 keep 1-66

PubMed search strategy

#4 Search: #1 AND #2 AND #3 Filters: Clinical Trial, Meta-Analysis, Randomized Controlled Trial, Systematic Review, in the last 1 year, Humans
#3 Search: renal OR kidney OR nephropath* Filters: Clinical Trial, Meta-Analysis, Randomized Controlled Trial, Systematic Review, in the last 1 year, Humans
#2 Search: diabet* Filters: Clinical Trial, Meta-Analysis, Randomized Controlled Trial, Systematic Review, in the last 1 year, Humans
#1 Search: ketoanalog* OR keto-analog* OR 'ketoacid supplements' or ketoacids OR 'keto acids' Filters: Clinical Trial, Meta-Analysis, Randomized Controlled Trial, Systematic Review, in the last 1 year, Humans

Web of Science search strategy

#1 25 TOPIC: (ketoanalog* OR keto-analog* OR 'ketoacid supplements')
AND TOPIC: (diabet*) AND TOPIC: (renal or kidney or nephropath*)
Indexes=SCI-EXPANDED, SSCI, A&HCI, CPCI-S, CPCI-SSH, ESCI, CCR-EXPANDED, IC
Timespan=All years

Supplementary materials to the manuscript: "**Ketoanalogue Supplementation in Patients with Non-dialysis Diabetic Kidney Disease: A Systematic Review and Meta-analyses**" by V. Bellizzi et al.

Figure S1. Funnel plots displaying results of analyses for potential publication bias for the meta-analyses

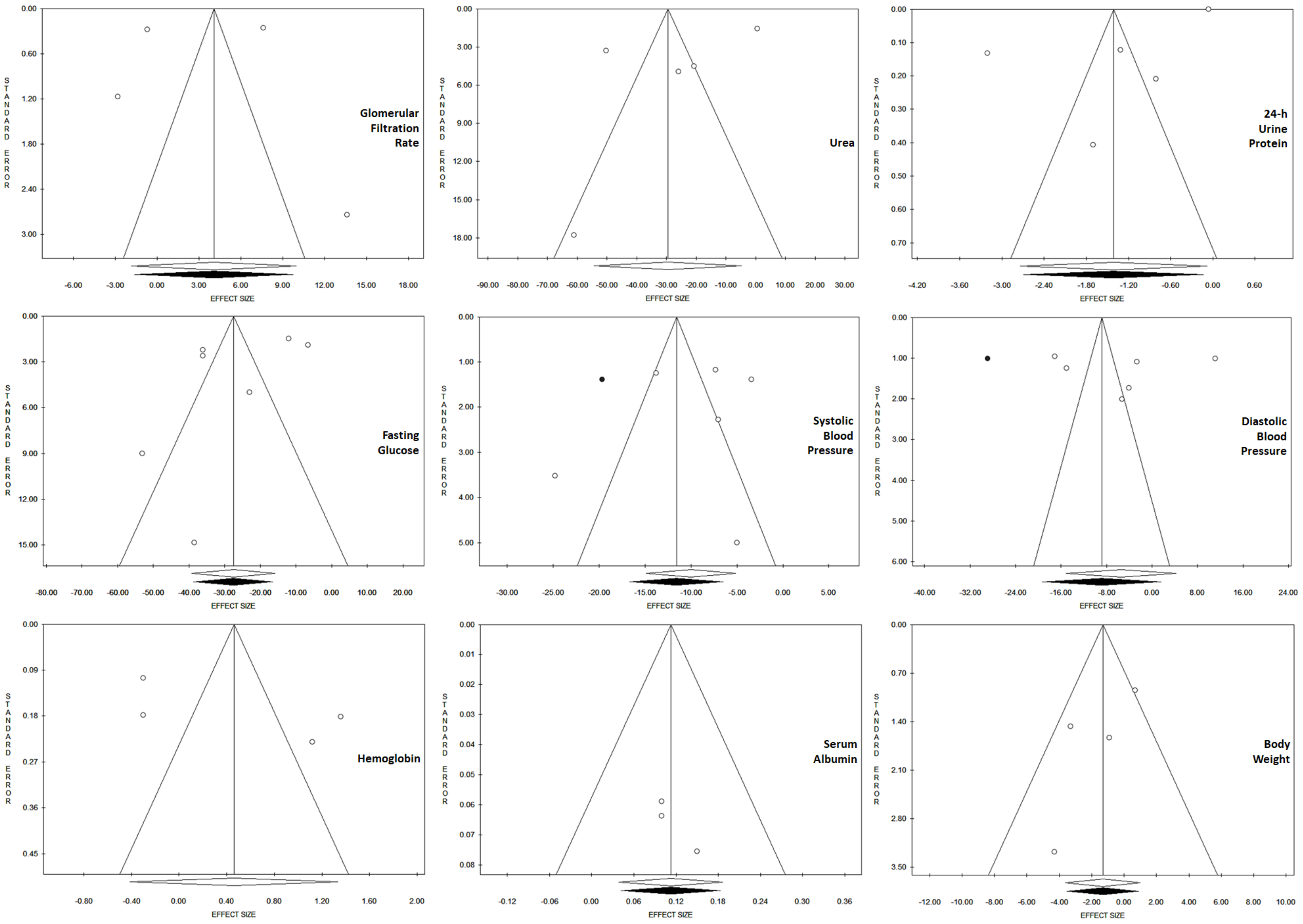

Table S1. Methodological quality assessment of randomized controlled trials.[1]

	Alam et al [29]			Khan et al [12]			Zhu et al [36]		
Criteria	Yes	No	NR/NA	Yes	No	NR/NA	Yes	No	NR/NA
Was the study described as randomized, a randomized trial, a randomized clinical trial, or an RCT?	X			X			X		
Was the method of randomization adequate (i.e., use of randomly generated assignment)?	X			X					NR
Was the treatment allocation concealed so that assignments could not be predicted?			NR		X				NR
Were study participants and providers blinded to treatment group assignment?		X		X				X	
Were the people assessing the outcomes blinded to the participants' group assignments?		X		X				X	
Were the groups similar at baseline on important characteristics that could affect outcomes?		X		X			X		
Was the overall drop-out rate from the study at endpoint ≤20% of the number allocated to treatment?	X			X			X		
Was the differential drop-out rate (between treatment groups) at endpoint ≤15 percentage points?	X			X			X		
Was there high adherence to the intervention protocols for each treatment group?			NR			NR			NR
Were other interventions avoided or similar in the groups (e.g., similar background treatments)?		X		X			X		
Were outcomes assessed using valid and reliable measures, implemented consistently across all study participants?	X			X				X	
Did the authors report that the sample size was sufficiently large to detect a difference in the main outcome between groups with ≥80% power?		X			X			X	
Were all reported outcomes and subgroup analyses prespecified in the study protocol?		X			X			X	
Were all randomized participants analyzed using an intention-to-treat analysis?		X			X		X		
Quality rating (good, fair, poor)	Fair			Fair			Fair		

NA, not applicable; NR, not reported; RCT, randomized controlled trial

[1] Reported as the consensus rating for each domain.

Table S2. Methodological quality assessment of observational cohort and cross-sectional studies.[1]

	Bellizzi et al [2]			Teodoru et al [34]			Wang et al [35]			Chen et al [32]		
Criteria	Yes	No	NR/ NA	Yes	No	NR/ NA	Yes	No	NR/ NA	Yes	No	NR/ NA
Was the research question or objective in this paper clearly stated?	X				X		X			X		
Was the study population clearly specified and defined?	X			X			X			X		
Was the participation rate of eligible persons at least 50%?	X					NA	X			X		
Were all the subjects selected or recruited from the same or similar populations Were inclusion and exclusion criteria prespecified?	X					NR	X			X		
Was a sample size justification, power description, or variance and effect estimates provided?		X			X			X			X	
For the analyses in this paper, were the exposure(s) of interest measured prior to the outcome(s) being measured?			NR		X		X			X		
Was the timeframe sufficient so that one could reasonably expect to see an association between exposure and outcome if it existed?	X			X			X			X		
For exposures that can vary in amount or level, did the study examine different levels of the exposure as related to the outcome?			NA	X				X			X	
Were the exposure measures (independent variables) clearly defined, valid, reliable, and implemented consistently across all study participants?			NA		X			X			X	
Was the exposure(s) assessed more than once over time?			NA	X				X		X		

Were the outcome measures (dependent variables) clearly defined, valid, reliable, and implemented consistently across all study participants?	X			X			X			X		
Were the outcome assessors blinded to the exposure status of participants?			NA		X			X			X	
Was loss to follow-up after baseline 20% or less?		X				NA	X			X		
Were potential confounding variables measured and adjusted statistically for impact on the relationship between exposure(s) and outcome(s)?			NR		X		X			X		
Quality rating (good, fair, poor)	Good			Poor			Fair			Fair		

NA, not applicable; NR, not reported

[1] Reported as the consensus rating for each domain.

Table S3. Methodological quality assessment for pre-post studies with no control group.[1]

	Barsotti et al [30]			Barsotti et al [31]			Chang et al [8]			Mihalache et al [33]		
Criteria	Yes	No	NR/NA	Yes	No	NR/NA	Yes	No	NR/NA	Yes	No	NR/NA
Was the study question or objective clearly stated?		X			X		X			X		
Were eligibility/selection criteria for the study population prespecified and clearly described?		X			X			X		X		
Were the participants in the study representative of those who would be eligible for the test/service/intervention in the general or clinical population of interest?		X			X		X			X		
Were all eligible participants that met the prespecified entry criteria enrolled?		X				NR		X			X	
Was the sample size sufficiently large to provide confidence in the findings?		X			X			X				NR
Was the test/service/intervention clearly described and delivered consistently across the study population?		X		X			X			X		
Were the outcome measures prespecified, clearly defined, valid, reliable, and assessed consistently across all study participants?	X			X			X			X		
Were the people assessing the outcomes blinded to the participants' exposures/interventions?		X			X			X			X	
Was the loss to follow-up after baseline 20% or less? Were those lost to follow-up accounted for in the analysis?			NR			NR		X		X		
Did the statistical methods examine changes in outcome measures from before to after the intervention? Were statistical tests done that provided p values for the pre-to-post changes?	X			X			X		X			

Were outcome measures of interest taken multiple times before the intervention and multiple times after the intervention (i.e., did they use an interrupted time-series design)?	X			X			X			X		
If the intervention was conducted at a group level, did the statistical analysis account for the use of individual-level data to determine effects at the group level?			NA			NA			NA			NA
Quality rating (good, fair, poor)	Poor			Poor			Fair			Good		

NA, not applicable; NR, not reported

[1] Reported as the consensus rating for each domain.
